# Supplementary material for: Bactericidal Biodegradable Linear Polyamidoamines Obtained with the Use of Endogenous Polyamines
Source: Int J Mol Sci. 2024 Feb 22;25(5):2576. doi: 10.3390/ijms25052576 (PMC10931725; doi:10.3390/ijms25052576)
Supplement: Supplementary file 1 [file ijms-25-02576-s001.zip › ijms-2810440-supplementary.pdf]

International Journal of *Molecular Sciences*

MDPI

Supplementary Materials

Bactericidal biodegradable linear polyamidoamines obtained with the use of endogenous polyamines

Natalia Śmigiel - Gac[1]*, Anna Smola-Dmochowska[1], Katarzyna Jelonek[1], Monika Musiał-Kulik[1], Renata Barczyńska-Felusiak[2], Piotr Rychter[2], Kamila Lewicka[2] and Piotr Dobrzyński[1,2]*

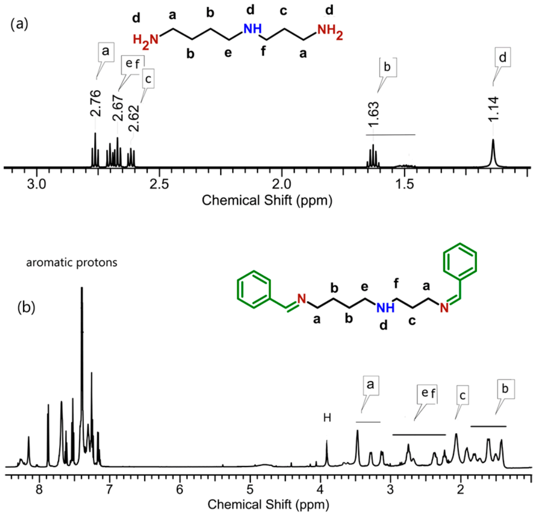

Figure S1. ^{1}H NMR (in $CDCl_3$) spectrum of spermidine (a) before protecting and (b) after deprotecting the primary amino groups.

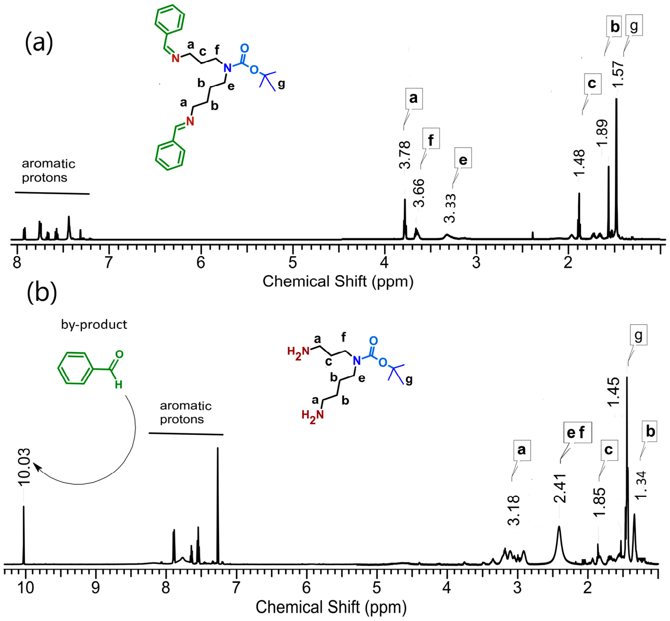

Figure S2. ^{1}H NMR (in $CDCl_3$) spectrum of a spermidine derivative with protected amino groups; (a) with protected primary and secondary amino groups, (b) after deprotecting primary amino groups (before cleaning).

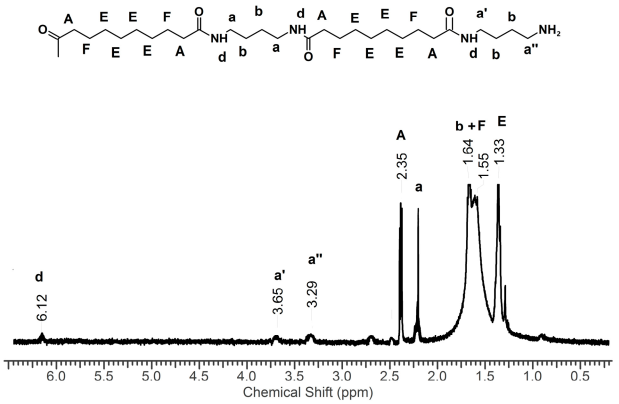

Figure S3. ^{1}H NMR (in $CDCl_3$) spectrum of polyamide obtained with putrescine.

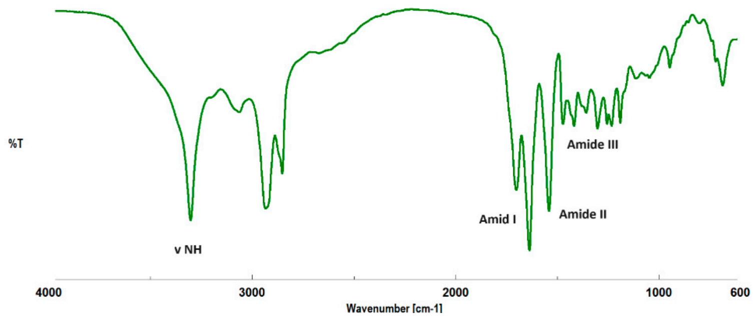

Figure S4. FTIR spectrum of polyamide obtained with putrescine.

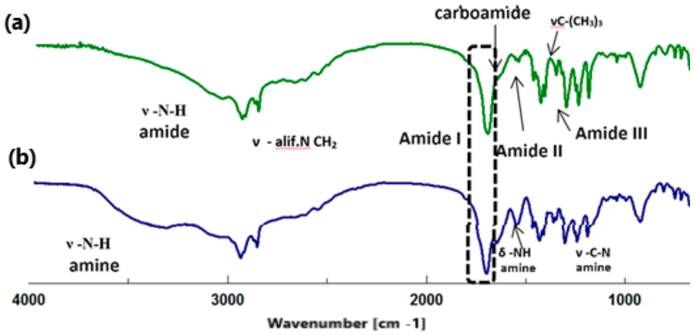

Figure S5. FTIR spectrum of polyamidoamine obtained with norspermidine: a) before deprotection and b) after deprotection of the secondary amino groups;

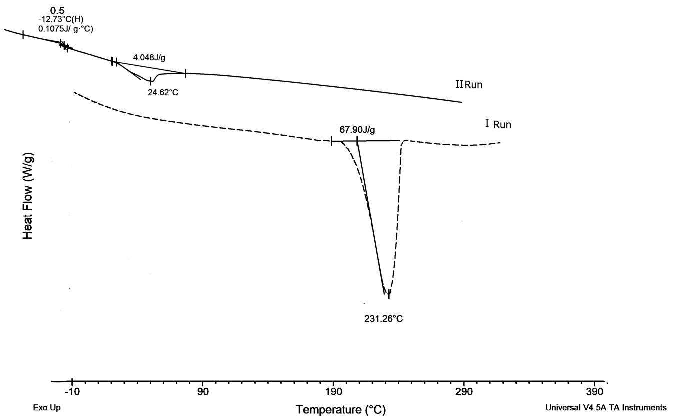

Figure S6. DSC thermograms (I and II run) of polyamide obtained with putrescine

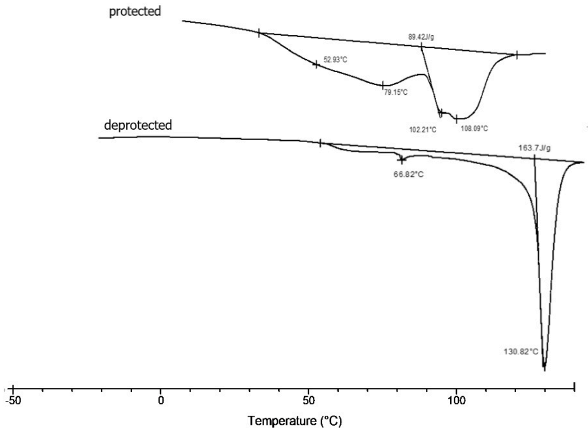

Figure S7. DSC thermograms of the run I, a polyamidoamine obtained using norspermidine with protected and deprotected amino groups.

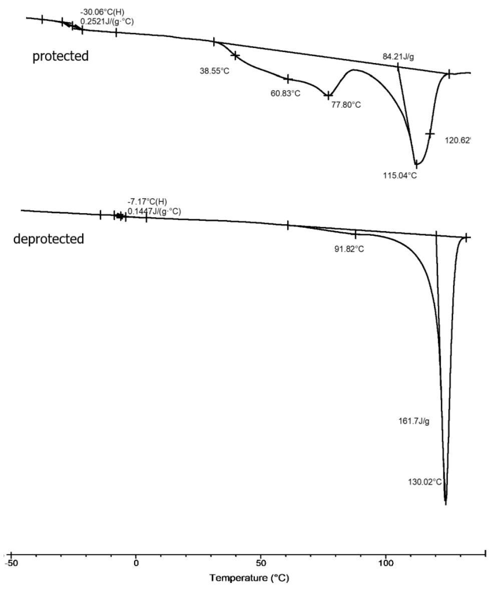

Figure S8. DSC thermograms of run I, a polyamidoamine obtained using spermidine with protected and deprotected amino groups.

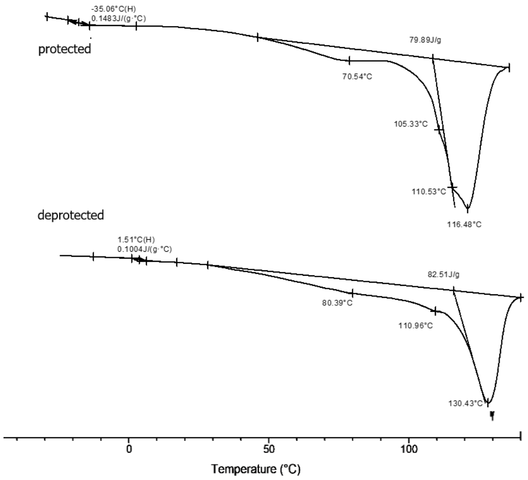

Figure S9. DSC thermograms of run I, a polyamidoamin obtained using spermine with protected and deprotected amino groups.

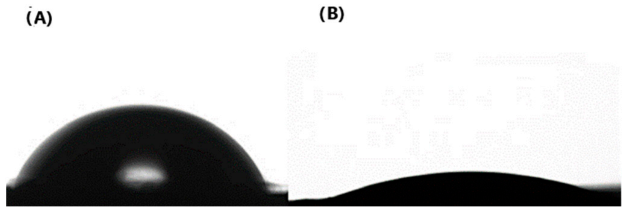

Figure S10. Measurement of the contact angle of the surface of a film cast from polyamidoamine obtained with norspermidine (A) with protected amino groups, (B) after deprotection of amino groups.

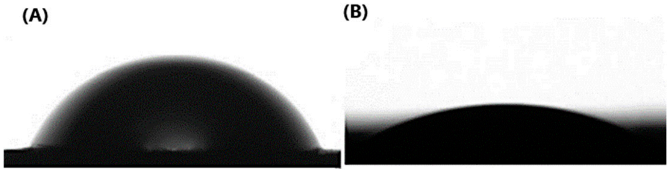

Figure S11. Measurement of the contact angle of the surface of a film cast from polyamidoamine obtained with spermidine (A) with protected amino groups, (B) after deprotection of amino groups.

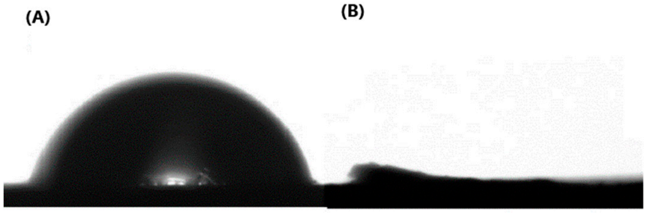

Figure S12. Measurement of the contact angle of the surface of a film cast from polyamidoamine obtained with spermine (A) with protected amino groups, (B) after deprotection of amino groups.
